# The Effects of a TMS Double Perturbation to a Cortical Network

**DOI:** 10.1523/ENEURO.0188-19.2019

**Published:** 2020-01-29

**Authors:** Ian G. M. Cameron, Andreea L. Cretu, Femke Struik, Ivan Toni

**Affiliations:** Donders Institute for Brain Cognition and Behaviour, Centre for Cognitive Neuroimaging, Radboud University Nijmegen, 6525 EN, Nijmegen, The Netherlands

**Keywords:** FEF, frontal eye fields, parietal cortex, prefrontal cortex, saccade, transcranial magnetic stimulation

## Abstract

Transcranial magnetic stimulation (TMS) is often used to understand the function of individual brain regions, but this ignores the fact that TMS may affect network-level rather than nodal-level processes. We examine the effects of a double perturbation to two frontoparietal network nodes, compared with the effects of single lesions to either node. We hypothesized that Bayesian evidence for the absence of effects that build upon one another indicates that a single perturbation is consequential to network-level processes. Twenty-three humans performed pro-saccades (look toward) and anti-saccades (look away) after receiving continuous theta-burst stimulation (cTBS) to right frontal eye fields (FEFs), dorsolateral prefrontal cortex (DLPFC), or somatosensory cortex (S1; the control region). On a subset of trials, a TMS pulse was applied to right posterior parietal cortex (PPC). FEF, DLPFC, and PPC are important frontoparietal network nodes for generating anti-saccades. Bayesian *t* tests were used to test hypotheses for enhanced double perturbation effects (cTBS plus TMS pulse) on saccade behaviors, against the alternative hypothesis that double perturbation effects to a network are not greater than single perturbation effects. In one case, we observed strong evidence [Bayes factor (BF_10_) = 325] that PPC TMS following DLPFC cTBS enhanced impairments in ipsilateral anti-saccade amplitudes over DLPFC cTBS alone, and not over the effect of the PPC pulse alone (BF_10_ = 0.75), suggesting that double perturbation effects do not augment one another. Rather, this suggests that computations are distributed across the network, and in some cases there can be compensation for cTBS perturbations.

## Abstract (precis)

Transcranial magnetic stimulation (TMS) is often used to understand the function of individual brain regions, but this ignores the fact that TMS may affect network-level rather than nodal-level processes. We examine the effects of a double perturbation to two frontoparietal network nodes, compared with the effects of single lesions to either node.

## Significance Statement

We explore whether a frontoparietal network important to executive control, attentional processing, and saccadic gaze behaviors operates in a distributed fashion, compared with what would be predicted from combining contributions from individual brain regions. This is important as lesions or perturbations to these regions individually can produce behavioral deficits. We apply inhibitory transcranial magnetic stimulation (TMS) to a frontal cortical region, followed by a second TMS perturbation to a parietal region. The point is that this second perturbation could, in principle, build on the effects of the first perturbation. We tested different hypotheses regarding the effects of such double perturbations and conclude that the effects do not build on one another, suggesting that a single perturbation affects a network-level process.

## Introduction

It is well known that the effects of transcranial magnetic stimulation (TMS) extend beyond the site of stimulation (Ilmoniemi et al., 1997; Paus et al., 1997; Ruff et al., 2006; Ko et al., 2008; Morishima et al., 2009). In some instances, distal effects may reflect compensatory responses to the TMS perturbation (Sack et al., 2005; O’Shea et al., 2007; Hartwigsen et al., 2013), suggesting “homeostatic metaplasticity” (Müller-Dahlhaus and Ziemann, 2015) at the level of network nodes. Here we assess another functionally relevant possibility: whether behavioral consequences of a spatially localized perturbation from TMS are driven by the distributed nature of computations throughout a circuit (Price and Friston, 2002). This would have consequences as to whether nodal effects build on one another.

The saccadic eye-movement system provides a tractable testing ground for assessing circuit-level consequences of TMS (Leigh and Kennard, 2004; Munoz et al., 2007). Roles of three cortical nodes, frontal eye fields (FEFs), dorsolateral prefrontal cortex (DLPFC), and posterior parietal cortex (PPC) have been well described (Munoz and Everling, 2004; Johnston and Everling, 2011; Paré and Dorris, 2011). In the anti-saccade task (where subjects must look away from a peripheral visual stimulus; Hallett, 1978), DLPFC is thought to be critical to establishing the appropriate task set and preventing an automatic saccade to the stimulus; FEF is thought to be critical to voluntary saccade programming and to “preparatory set”; and FEF along with PPC are thought to be critical to the visuo-motor transformations to develop a saccade “vector” (Connolly et al., 2002; Leigh and Kennard, 2004; Munoz and Everling, 2004).

Evidence shows how DLPFC, FEF, and PPC interact as part of a distributed system, as follows: TMS to either DLPFC or FEFs (or supplementary eye fields) during saccade programming prolonged reaction times, suggesting preparatory set is distributed among all three nodes (Nagel et al., 2008). Magnetoencephalography (MEG) and functional magnetic resonance imaging (fMRI) showed that FEF and PPC are both involved in the attentional aspects of the anti-saccade vector (Medendorp et al., 2005; Moon et al., 2007), and TMS to FEF or PPC produces hypometric anti-saccades (Nyffeler et al., 2008b; Jaun-Frutiger et al., 2013; Cameron et al., 2015). However, it is not possible to distinguish a difference in timing (even with MEG) between when an anti-saccade program is developed in the PPC compared with FEF (Moon et al., 2007), implying a distributed process.

**Figure 1.**
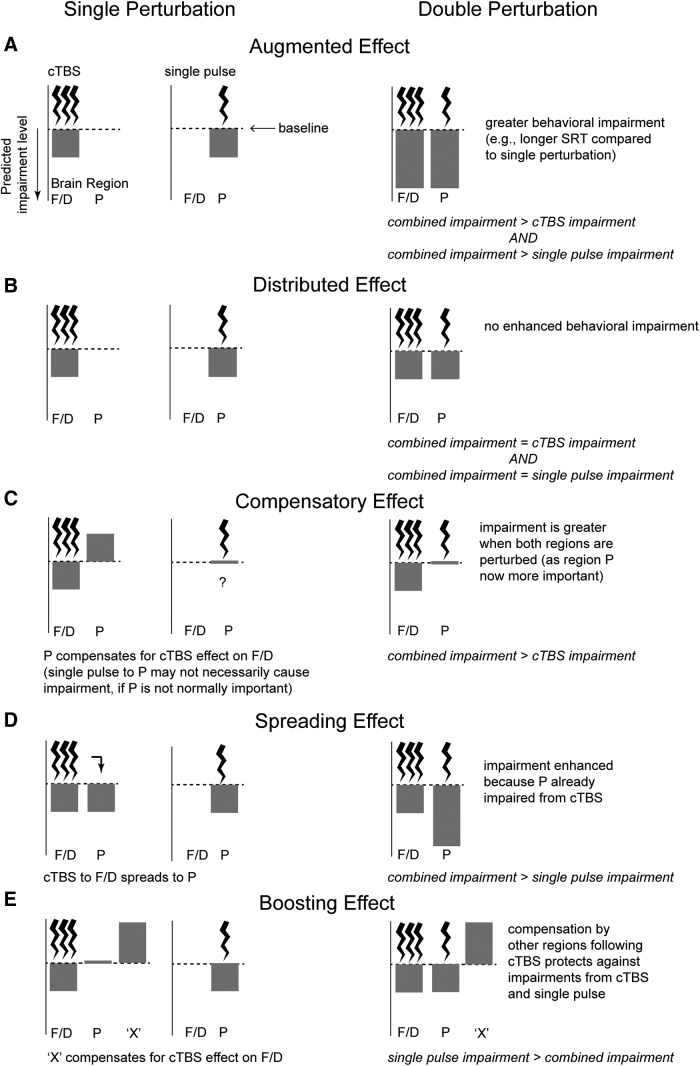
Hypotheses for the effects of TMS perturbations to two oculomotor network nodes (e.g., F, frontal eye fields; D, dorsolateral prefrontal cortex; and P, posterior parietal cortex) in the same hemisphere. ***A***, Augmented: augmented impairment from a double perturbation compared with a single perturbation to either node. ***B***, Distributed: no augmented effects (a single perturbation to the network is equally disruptive). ***C***, Compensatory: compensatory effect from second node that became more important. ***D***, Spreading: greater effect due to cTBS spreading through the network to influence the second node. ***E***, Boosting: additional network regions (region “X”) provide sources of compensation after cTBS leading to a boost to performance.

We build on this knowledge to study the effects on behavior after a “double perturbation” to this network in the right hemisphere. Shortly after applying continuous theta-burst stimulation (cTBS; Huang et al., 2005) to either right FEF (r-FEF) or right DLPFC (r-DLPFC), we measure the consequences of a second time-resolved perturbation to the circuit, in the form of a single TMS pulse to right PPC (r-PPC). This approach arbitrates among five hypotheses regarding the consequences of the double perturbation. In hypothesis A, “Augmented,” the double perturbation could produce an augmented effect by concurrently impairing spatially separate nodes that provide critical, but computationally distinct, functions, resulting in behavioral perturbations that are greater than the effect of either perturbation alone (Fig. 1*A*). Alternatively, hypothesis B, “Distributed,” pertains to the case where computations are performed by a distributed system at the network level, so a single perturbation to either node should perturb behavior as much as the double perturbation (Price et al., 2017; Fig. 1*B*). In hypothesis C, “Compensatory,” distal nodes could compensate for the perturbation, which would predict greater effects from the double perturbation compared with the cTBS perturbation alone (Fig. 1C), because the second perturbation impairs a region that has become more important functionally, because of the first (cTBS) perturbation. In hypothesis D, “Spreading,” the effects from cTBS spread trans-synaptically to other portions of the network (Ko et al., 2008), predicting greater effects from the double perturbation than from the single-pulse perturbation alone (Fig. 1*D*). Finally, in hypothesis E, “Boosting,” additional regions throughout the network could provide homeostatic compensation, which would manifest as a perplexing boost to performance following cTBS (alone) and could reduce or prevent the impairment from additional TMS perturbations (Fig. 1*E*). (The difference between this and the Compensatory hypothesis is that there is the perplexing boost to performance after cTBS.)

To discriminate among those hypotheses, we used fMRI to localize right DLPFC, FEF, and PPC in individual subjects performing an anti-saccade task. These regions were then used for targeting subject-specific TMS interventions while participants performed the same task outside the scanner. Performance (percentage correct direction), reaction times, and saccade amplitudes were assessed using Bayesian *t* tests to provide statistical evidence in favor or against greater effects from double-TMS, compared with single-TMS, perturbations.

## Materials and Methods

### Participants

The study was approved by the local ethics committee (Commissie Mensgebonden Onderzoek, Arnhem-Nijmegen), and written informed consent was obtained from the participants in accordance with the Declaration of Helsinki. A total of 27 healthy, right-handed, young-adult, human subjects was recruited for four sessions ∼1 week apart. Three subjects were excluded for failure to provide usable eye-tracking data on all TMS sessions, and one subject had error rates on anti-saccade trials exceeding 90% (>3× the SD) and so was excluded, resulting in a sample size of 24 participants (mean ± SE age, 23 ± 2 years; 11 males).

### Detailed procedure

#### Session 1

Participants were screened for contraindications related to fMRI and to single-pulse TMS and cTBS according to common safety guidelines (Rossi et al., 2009; Oberman et al., 2011). Resting motor thresholds (RMTs) and active motor thresholds (AMTs) were established for the first dorsal interosseous muscle of the subject’s right hand using electromyography. TMS was applied using a hand-held biphasic figure-eight coil with a 75 mm outer winding diameter (MagVenture), connected to a MagProX100 System (MagVenture). Coil orientation was chosen to induce a posterior–anterior electrical field in the brain (45° from the mid-sagittal axis).

**Figure 2.**
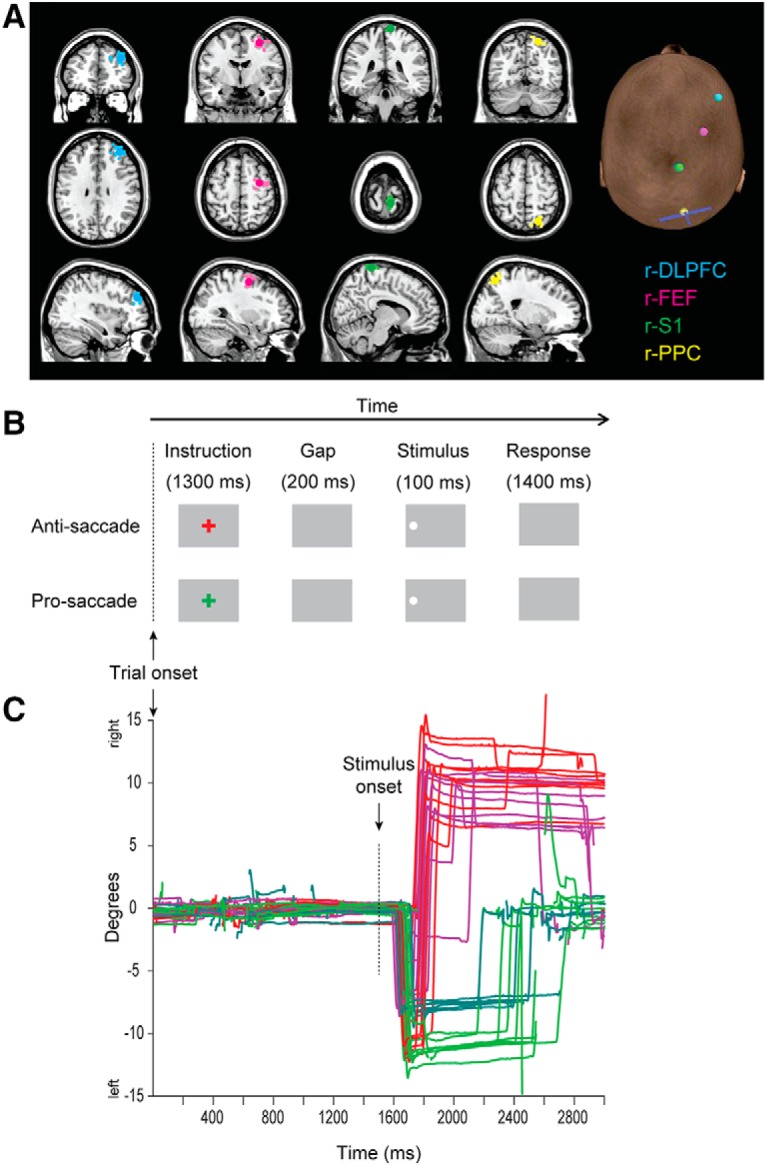
***A***, MRI images: illustration of coil placement over r-DLPFC, r-FEFs, r-S1, and r-PPC on an SPM single-subject anatomic template. Mean coordinates are shown as large bright dots, and individual subject coordinates are shown as faint dots. Right, Scalp “entry” points for TMS stimulation for a representative subject, showing also a representation of the coil orientation over right PPC (handle of coil = base of “T” shape). ***B***, Paradigm and stimulus timings shown for representative anti-saccade and pro-saccade trials, where the target stimulus was on the left side. ***C***, Illustrations of raw eye-traces from a representative subject in one run (subject 22841) with respect to stimuli on the left side. For 13° stimuli, red illustrates anti-saccades and green illustrates pro-saccades; for 9° stimuli, magenta illustrates anti-saccades, and turquoise illustrates pro-saccades. This subject made a high proportion of direction errors on anti-saccade trials in this run, indicated by the reversals of direction. Blinks are shown as gaps in the traces.

Subjects performed five runs of an interleaved pro-saccade (look toward)/anti-saccade (look away) task to identify the cortical regions of interest (ROIs; Fig. 2*B*). An interleaved task was used as evidence suggests an important role for DLPFC (Everling and DeSouza, 2005; Johnston et al., 2014) as well as for FEF (DeSouza and Everling, 2010) in task or preparatory set and thus could not simply default to an anti-saccade task set on each trial. Two target positions (13° or 9°) in the left and right directions were included so that subjects would have to rely on spatial information to calculate the saccade vector. In this way, we could be sure that the paradigm required DLPFC, FEF, and PPC processes.

#### Detailed fMRI procedure

fMRI scans were obtained with a 3 tesla MRI scanner (Skyra, Siemens Medical Systems) using a 32-channel head coil. The functional images were acquired with multiband sequence [acceleration factor = 3, repetition time (TR) = 1000 ms, echo time (TE) = 30 ms, flip angle = 60°). Each volume consisted of 33 slices, with a distance of 17% and a thickness of 3 mm. The voxel resolution was 3.5 × 3.5 × 3.0 mm, the FOV in the read direction was 224 mm, and the FOV in the phase direction was 100%. Two volumes were discarded from each functional run to account for scanner steady-state equilibrium, leading to a total of 339 volumes per run. The anatomic images were acquired with an MPRAGE sequence (TR = 2300 ms, TE = 3.9 ms, voxel size = 1 × 1 × 1 mm). In total, 192 images were obtained for each participant. During the scan, participants lay in a supine position and their heads were stabilized using soft cushions.

Imaging data were analyzed with SPM8 (Wellcome Trust Center for Cognitive Neuroimaging, London, UK). At the single-subject level, the data were realigned to the first volume of each run using six rigid body transformations (three translations and three rotations). The images were then coregistered to the individual structural T1, and spatial smoothing was performed by means of an 8 mm full-width at half-maximum Gaussian kernel. A first-level analysis was performed by specifying a general linear model with regressors for each condition (fixation trials were not modeled, however). Motion parameters (three translations, three rotations) were included as nuisance regressors.

A contrast of anti-saccade trials against baseline was computed to define 5 mm ROIs centered on locations of peak activation on each subject anatomic scan, using a *t* contrast at *p* < 0.001 (uncorrected). Table 1 provides the Montreal Neurologic Institute (MNI) coordinates of these ROIs, and their distances to the scalp as derived from Localite TMS Navigation software 2.2. Figure 2*A* illustrates the coordinates on a canonical T1 scan. r-DLPFC was defined as peak fMRI anti-saccade activity surrounding the middle frontal gyrus, anterior to the ventricles. Right FEF was defined as peak activity in the precentral sulcus (selecting medial peaks if lateral peaks were also present, to relate more to anti-saccade processes; Neggers et al., 2012). r-PPC was defined as peak activity in the intraparietal sulcus, selecting peaks in more medial clusters if more than one was present. Finally, right S1 (r-S1; the control region) was localized anatomically for each participant, as the most superior extent of the postcentral gyrus, located on average 9 ± 2 mm lateral to the longitudinal fissure to avoid lateral proprioceptive eye-position signals ( Zhang et al., 2008; Balslev et al., 2011; Table 1, Fig. 2*A*).

**Table 1:** ROI information (average ± SD)

	Coordinates (MNI space)			
	*x*	*y*	*z*	Distance to scalp (mm)
r-DLPFC	35 ± 7	45 ± 10	31 ± 7	19 ± 4
r-FEF	30 ± 5	−5 ± 4	57 ± 6	26 ± 5
r-PPC	20 ± 7	−66 ± 6	60 ± 5	22 ± 4
r-S1	9 ± 2	−38 ± 5	79 ± 2	20 ± 3

#### Session 2–4

cTBS was applied to r-DLPFC, r-FEF, or r-S1 before performing the task on three separate sessions, counterbalanced for order. cTBS was applied to FEF or to DLPFC because we wished to assess double perturbation effects across two nodes, which are both linked to PPC, but where one (FEF) is thought to have a more direct link in visuo-motor processes (Leigh and Kennard, 2004; Munoz and Everling, 2004) and in network interactions described in the resting state (Corbetta et al., 2005; He et al., 2007; Vossel et al., 2014). cTBS was delivered with a posterior–anterior direction of the electric field induced in the brain, with the handle pointed backward at ∼30° to the sagittal plane. In this way, the outer windings of the TMS coil did not overlap the other ROIs. TMS coil alignment was achieved using Localite and a subject-specific anatomic scan.

The parameters for cTBS were identical to those described by Huang et al. (2005) consisting of 50 Hz triplets repeated at 5 Hz over a period of 40 s. Stimulation intensity for cTBS was defined as 80% of the AMT (mean = 41% ± 9% maximum stimulator output), defined as peak-to-peak motor-evoked potential (MEP) amplitudes exceeding 200 μV on 5 of 10 trials, while subjects maintained voluntary contraction of ∼10%. Stimulation intensity for single-pulse TMS to PPC was set at 110% of the RMT (mean = 43% ± 8% maximum stimulator output), defined as peak-to-peak MEP amplitudes of 50 μV on 5 of 10 trials. Forty seconds of cTBS (at 80% of AMT) has effects lasting ∼50 min (Wischnewski and Schutter, 2015), providing sufficient time to test the influence of the PPC pulse.

##### Eye tracking and task

The position of the right eye was recorded using an infrared Eyelink 1000 eye tracker (SR Research) with a 1000 Hz sampling rate. A 9-point calibration was conducted, and a drift correction point was used as the intertrial fixation point. Saccades were identified by a horizontal deflection (3× SDs of the baseline velocity) and a duration between 15 and 150 ms. The camera was positioned under the stimulus screen, ∼60 cm away from the eyes of the participant, who sat precisely at 70 cm from a wide-angle LCD screen (with central presentation zone set at 4:3, and 1024 × 768 resolution).

Subjects performed the same task (Fig. 2*B*) as in the fMRI. Representative eye traces from a single subject are shown in Figure 2*C*. In each run, there were 72 trials, of which 48 contained a TMS pulse presented to PPC at a random interval between 30 and 300 ms after the onset of the peripheral stimulus (described in Data analysis). The first run commenced 10 min after cTBS and was analyzed up to 50 min after cTBS to capture the same cTBS effects on each session. Subjects were asked to perform five runs, each taking ∼8 min, including drift corrections and breaks, meaning that for each condition of interest (task, direction) there were 30 trials without the single pulse (“pulse absent”), and 60 trials containing the single pulse.

### Data analysis

Data were analyzed in MATLAB version 11 (MathWorks). Valid trials consisting of correct and incorrect directions were separated from invalid trials, consisting of saccade reaction times (SRTs) < 90 ms (anticipatory errors), slower than 1000 ms, and trials where the TMS pulse to PPC occurred after saccade onset. The following three behavioral parameters of interest were analyzed: amplitude of the primary saccade, percentage correct direction, and SRT.

**Figure 3.**
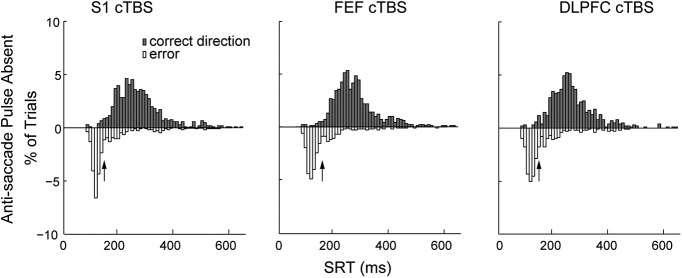
Derivation of the early and late PPC pulse bins based on anti-saccade reaction times. Reaction time distributions were calculated for correct and direction error anti-saccades in PPC pulse absent trials on each cTBS session. A binomial sign test was performed that compared the distributions, and arrows indicate the first reaction time bin where the two distributions were no longer significantly different. This value was taken as the boundary for early and late PPC pulses.

We first set a division between an “Early” and “Late” pulse time bin as follows: using the pulse absent trials, we collected the SRTs across subjects for correctly performed anti-saccades and for direction errors on anti-saccades for each cTBS session separately, and plotted these data in 10 ms bin histograms (Fig. 3). A binomial test revealed the first bin (Fig. 3, black arrows) where the two trial types were no longer significantly different from chance (50%); these bins occurred at 150 ms for the S1 cTBS and DLPFC cTBS sessions, and at 160 ms for the FEF-cTBS session. This method approximates the division between visually triggered “express” pro-saccades and voluntary saccades (Munoz and Everling, 2004), and is important to approximate when the PPC pulse would have greater influences during visual processing rather than motor programming components of an anti-saccade, which are in different directions.

We performed a repeated-measures ANOVA in SPSS (IBM) using pulse absent trials to determine whether there were significant interactions between the site of cTBS and stimulus eccentricity for amplitudes. However, no interactions with cTBS site and eccentricity were significant (*F*_(2,44)_ *<* 1.75, *p* > 0.19), so we collapsed across eccentricity. Next, we performed a multivariate repeated-measures ANOVA using pulse absent trials, split into the first and second half of testing time, to examine whether there were any significant interactions involving Half and cTBS Site across the three parameters of interest (a potential concern being that cTBS effects wore off): however, no interactions with cTBS Site and Half reached significance (Pillai’s trace values < 0.19, *F*_(6,86)_ < 1.54, *p* > 0.18).

### Statistics

**Figure 4.**
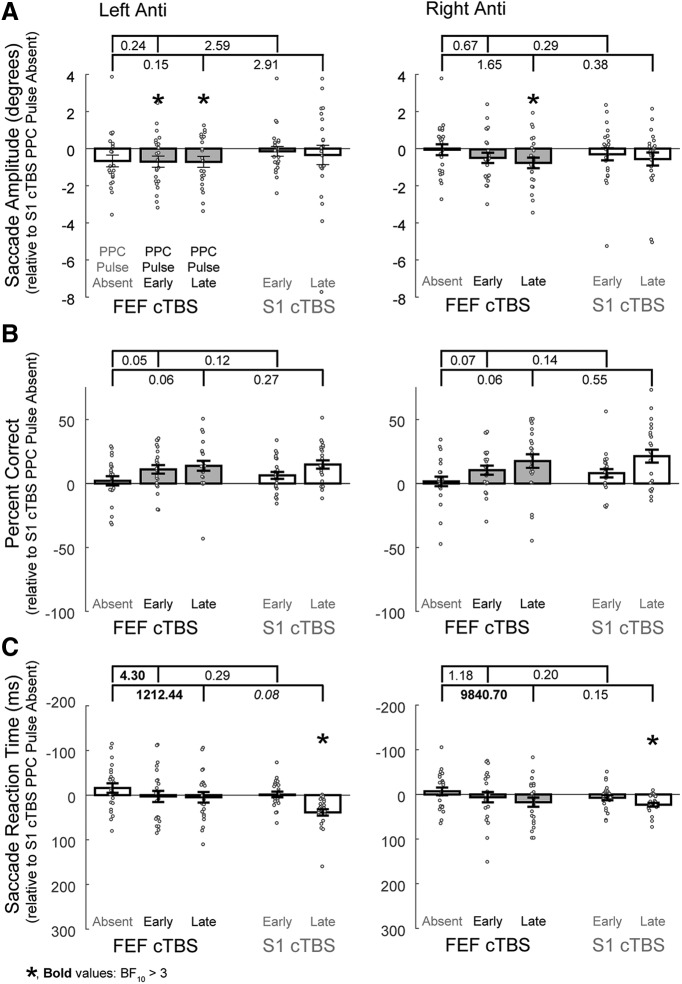
Effects on left and right anti-saccades when the double perturbation involving FEF cTBS and PPC TMS are compared with the single perturbation conditions for ***A***, Saccade amplitudes, ***B***, Percentage correct directions, and ***C***, Saccade reaction times. All data are normalized to the cTBS control condition (cTBS to S1, no PPC pulses). Error bars represent SEM across subjects (*N* = 23), and dark gray represents the double perturbation conditions. Values between brackets indicate the Bayes factor evidence for the alternative hypothesis that the combined effects from the double perturbation resulted in a greater impairment (more negative values, note the *y*-axis is reversed for saccade reaction times) compared with the effects of the single perturbations. Values >3 provide substantial evidence for the alternative hypothesis that the combined effects resulted in a greater impairment than the single perturbation effects. Asterisks show the results from Bayesian one-sample *t* tests for evidence that the values are <0 for amplitude and percentage correct, or >0 for reaction time, where BF_10_ > 3.

To directly assess our five network hypotheses regarding the combined effects from cTBS and the PPC pulse (Fig. 1), we performed Bayesian paired-sample *t* tests in JASP (JASP Team, 2017; Figs. 4-Figs. 7, brackets). A Bayes factor (BF_10_) indicates the evidence for the alternative hypothesis relative to the null hypothesis given the data. Our tests were focused first on situations where the double perturbation produces impairments that were greater than the single perturbations; thus, BF_10_ here indicates whether the combined effects were greater than the individual effects from cTBS alone, or from PPC TMS alone. For amplitude and percentage correct, lower values are indicative of greater impairments: therefore, the alternative hypothesis for BF_10_ is that the difference of the combined effect minus the single perturbation effect was <0, and the null hypothesis would be that this difference is not less than zero. For reaction times, higher values are indicative of an impairment (slower latency), so the alternative hypothesis is that the combined effect minus the single perturbation effect is greater than zero (and the null hypothesis is that it is not greater than zero). Note, however, that strong evidence from these tests for the null hypothesis (not less than zero) could be driven by a difference in the opposite direction. When such “strong” evidence was found (BF_10_ < 0.1; Jeffreys, 1961; Wetzels et al., 2011), we subsequently performed tests in the opposite direction to determine whether the effect of the single perturbation was greater than that of the double perturbation.

We report evidence for behavioral impairments that meet or exceed “substantial” (BF_10_ > 3; Jeffreys, 1961; Wetzels et al., 2011). Between 0.33 and 3, the evidence is considered only “anecdotal,” and in relation to *p* values, it was shown that ∼70% of “positive” results from 855 tests falling in the interval between *p* < 0.01 to *p* < 0.05 corresponded to only anecdotal evidence (Wetzels et al., 2011). Therefore, our boundary criteria of substantial are conservative in relation to typical *p* values.

Tests for each individual trial type compared with the control condition (S1 cTBS, PPC pulse absent) were also conducted using Bayesian one-sample *t* tests in JASP to confirm whether the individual perturbations themselves caused impairments. Here, BF_10_ indicates the relative likelihood that cTBS or single-pulse TMS impaired behavior compared with the null hypothesis that the behaviors were not impaired relative to the control condition. The values for these tests are seen in Tables 3-Tables 6 and are seen as asterisks in Figures 4-Figures 7 when substantial.

Table 2 (statistics table) lists all BF values from the Bayesian *t* tests along with their corresponding effect sizes as the medians of the posterior distributions, with 95% confidence intervals.

**Table 2:** Statistical table

	Data structure	Type of test	BF_10_	Effect size: Median of posterior distribution [95% CI]	
Table 3, Amplitude					
L.A., F., Absent	Assumed normal	Bayesian *t* test	2.69	−0.40 [−0.82, −0.06]	
L.A., F., Early			4.09	−0.45 [−0.86, −0.08]	
L.A., F., Late			4.58	−0.47 [−0.90, −0.09]	
L.A., S., Early			0.35	−0.18 [−0.53, −0.01]	
L.A., S., Late			0.39	−0.19 [−0.54, −0.01]	
R.A., F., Absent			0.26	−0.15 [−0.47, −0.01]	
R.A., F., Early			1.31	−0.32 [−0.72, −0.03]	
R.A., F., Late			7.60	−0.51 [−0.94, −0.11]	
R.A., S., Early			0.52	−0.22 [−0.58, −0.02]	
R.A., S., Late			1.21	−0.31 [−0.71, −0.03]	
Table 3, Percentage correct					
L.A., F., Absent			0.15	−0.10 [−0.37, 0.00]	
L.A., F., Early			0.06	−0.03 [−0.12, 0.00]	
L.A., F., Late			0.06	−0.06 [−0.18, −0.01]	
L.A., S., Early			0.08	−0.06 [−0.22, 0.00]	
L.A., S., Late			0.05	−0.05 [−0.24, 0.00]	
R.A., F., Absent			0.16	−0.11 [−0.38, −0.01]	
R.A., F., Early			0.07	−0.05 [−0.22, 0.00]	
R.A., F., Late			0.06	−0.04 [−0.14, 0.00]	
R.A., S., Early			0.07	−0.06 [−0.22, 0.00]	
R.A., S., Late			0.06	−0.10 [−0.13, −0.01]	
Table 3, Saccade reaction time					
L.A., F., Absent			0.10	0.07 [0.00, 0.28]	
L.A., F., Early			0.27	0.15 [0.01, 0.47]	
L.A., F., Late			0.31	0.17 [0.01, 0.51]	
L.A., S., Early			0.19	0.12 [0.01, 0.40]	
L.A., S., Late			2299.54	1.04 [0.52, 1.59]	
R.A., F., Absent			0.13	0.09 [0.00, 0.35]	
R.A., F., Early			0.35	0.18 [0.01, 0.52]	
R.A., F., Late			1.39	0.33 [0.03, 0.74]	
R.A., S., Early			0.79	0.27 [0.02, 0.65]	
R.A., S., Late			2619.87	1.05 [0.53, 1.60]	
Table 4, Amplitude					
L.A., D., Absent			4.21	−0.45 [−0.88, −0.08]	
L.A., D., Early			0.55	−0.23 [−0.59, −0.02]	
L.A., D., Late			4.84	−0.47 [−0.89, −0.08]	
R.A., D., Absent			0.33	−0.17 [−0.51, −0.01]	
R.A., D., Early			0.98	−0.29 [−0.67, −0.03]	
R.A., D., Late			8.84	−0.52 [−0.96, −0.12]	
Table 4, Percentage correct					
L.A., D., Absent			0.27	−0.15 [−0.47, −0.01]	
L.A., D., Early			0.14	−0.09 [−0.35, 0.00]	
L.A., D., Late			0.05	−0.01 [−0.01, −0.01]	
R.A., D., Absent			0.16	−0.11 [−0.38, −0.01]	
R.A., D., Early			0.09	−0.07 [−0.25, 0.00]	
R.A., D., Late			0.05	−0.03 [−0.18, −0.01]	
Table 4, Saccade reaction time					
L.A., D., Absent			0.16	0.10 [0.01, 0.39]	
L.A., D., Early			0.11	0.07 [0.00, 0.31]	
L.A., D., Late			2.52	0.39 [0.06, 0.81]	
R.A., D., Absent			0.68	0.25 [0.02, 0.63]	
R.A., D., Early			0.33	0.17 [0.01, 0.51]	
R.A., D., Late			33.86	0.65 [0.22, 1.10]	
Table 5, Amplitude					
L.P., F., Absent			0.61	−0.23 [−0.60, −0.02]	
L.P., F., Early			0.31	−0.16 [−0.50, −0.01]	
L.P., F., Late			0.87	−0.28 [−0.66, −0.02]	
L.P., S., Early			0.11	−0.08 [−0.32, 0.00]	
L.P., S., Late			0.30	−0.16 [−0.50, −0.01]	
R.P., F., Absent			0.54	−0.23 [−0.59, −0.01]	

R.P., F., Early			0.56	−0.23 [−0.59, −0.01]	
R.P., F., Late			0.30	−0.16 [−0.51, −0.01]	
R.P., S., Early			1.07	−0.30 [−0.69, −0.03]	
R.P., S., Late			2.32	−0.39 [−0.83, −0.05]	
Table 5, Percentage correct					
L.P., F., Absent			0.12	−0.08 [−0.34, 0.00]	
L.P., F., Early			0.41	−0.20 [−0.56, −0.01]	
L.P., F., Late			2.63	−0.40 [−0.81, −0.06]	
L.P., S., Early			0.48	−0.21 [−0.56, −0.01]	
L.P., S., Late			1.44	−0.34 [−0.74, −0.04]	
R.P., F., Absent			1.16	−0.31 [−0.69, −0.03]	
R.P., F., Early			0.22	−0.13 [−0.43, −0.01]	
R.P., F., Late			4.53	−0.47 [−0.91, −0.09]	
R.P., S., Early			0.24	−0.14 [−0.46, −0.01]	
R.P., S., Late			1.19	−0.31 [−0.71, −0.03]	
Table 5, Saccade reaction time					
L.P., F., Absent			0.29	0.16 [0.01, 0.49]	
L.P., F., Early			14.878	0.57 [0.15, 1.02]	
L.P., F., Late			3314.92	1.08 [0.56, 1.63]	
L.P., S., Early			110.56	0.77 [0.30, 1.25]	
L.P., S., Late			52,637.20	1.40 [0.79, 2.03]	
R.P., F., Absent			0.16	0.10 [0.02, 0.38]	
R.P., F., Early			4.08	0.44 [0.08, 0.86]	
R.P., F., Late			1461.64	1.07 [0.53, 1.64]	
R.P., S., Early			51.42	0.69 [0.25, 1.16]	
R.P., S., Late			2,165,000.00	1.81 [1.09, 2.56]	
Table 6, Amplitude					
L.P., D., Absent			0.19	−0.12 [−0.41, 0.00]	
L.P., D., Early			0.24	−0.14 [−0.47, −0.01]	
L.P., D., Late			0.15	−0.10 [−0.36, −0.01]	
R.P., D., Absent			0.62	−0.24 [−0.62, −0.02]	
R.P., D., Early			0.22	−0.13 [−0.43, −0.01]	
R.P., D., Late			1.03	−0.29 [−0.69, −0.02]	
Table 6, Percentage correct					
L.P., D., Absent			0.22	−0.13 [−0.45, −0.01]	
L.P., D., Early			0.32	−0.17 [−0.50, −0.01]	
L.P., D., Late			0.42	−0.19 [−0.56, −0.01]	
R.P., D., Absent			0.17	−0.11 [−0.39, −0.01]	
R.P., D., Early			0.21	−0.13 [−0.44, −0.01]	
R.P., D., Late			2.84	−0.41 [−0.82, −0.06]	
Table 6, Saccade reaction time					
L.P., D., Absent			0.29	0.16 [0.01, 0.50]	
L.P., D., Early			3.65	0.43 [0.07, 0.85]	
L.P., D., Late			5089.32	1.12 [0.58, 1.67]	
R.P., D., Absent			0.12	0.09 [0.00, 0.33]	
R.P., D., Early			2.49	0.39 [0.05, 0.81]	
R.P., D., Late			2,344,000.00	1.75 [1.07, 2.46]	
Figure 4*A*					
L.A., F. Absent – F. Early			0.24	−0.14 [−0.45, −0.01]	
L.A., F. Early – S. Early			2.59	−0.40 [−0.80, −0.06]	
L.A., F. Absent – F. Late			0.15	−0.10 [−0.38, 0.00]	
L.A., F. Late – S. Late			2.91	−0.42 [−0.85, −0.06]	
R.A., F. Absent – F. Early			0.67	−0.25 [−0.62, −0.02]	
R.A., F. Early – S. Early			0.29	−0.16 [−0.49, −0.01]	
R.A., F. Absent – F. Late			1.65	−0.34 [−0.75, −0.05]	
R.A., F. Late – S. Late			0.38	−0.18 [−0.53, −0.01]	
Figure 4*B*					
L.A., F. Absent – F. Early			0.05	−0.05 [−0.25, 0.00]	
L.A., F. Early – S. Early			0.12	−0.08 [−0.34, 0.00]	
L.A., F. Absent – F. Late			0.06	−0.05 [−0.17, −0.01]	

L.A., F. Late – S. Late			0.27	−0.15 [−0.47, −0.01]	
R.A., F. Absent – F. Early			0.07	−0.05 [−0.23, 0.00]	
R.A., F. Early – S. Early			0.14	−0.10 [−0.35, 0.00]	
R.A., F. Absent – F. Late			0.06	−0.05 [−0.21, −0.01]	
R.A., F. Late – S. Late			0.55	−0.23 [−0.59, −0.02]	
Figure 4*C*					
L.A., F. Absent – F. Early			4.30	0.45 [0.09, 0.87]	
L.A., F. Early – S. Early			0.29	0.15 [0.01, 0.49]	
L.A., F. Absent – F. Late			1212.45	1.02 [0.49, 1.55]	
L.A., F. Late – S. Late			0.08	0.06 [0.00, 0.28]	
R.A., F. Absent – F. Early			1.18	0.31 [0.03, 0.71]	
R.A., F. Early – S. Early			0.204	0.12 [0.01, 0.44]	
R.A., F. Absent – F. Late			9840.70	1.17 [0.63, 1.75]	
R.A., F. Late – S. Late			0.152	0.10 [0.01, 0.38]	
Figure 5*A*					
L.A., D. Absent – D. Early			0.11	−0.14 [−0.45, −0.01]	
L.A., D. Early – S. Early			0.42	−0.40 [−0.80, −0.06]	
L.A., D. Absent – D. Late			0.49	−0.22 [−0.57, −0.02]	
L.A., D. Late – S. Late			0.64	−0.24 [−0.62, −0.02]	
R.A., D. Absent – D. Early			0.84	−0.25 [−0.62, −0.02]	
R.A., D. Early – S. Early			0.46	−0.16 [−0.49, −0.01]	
R.A., D. Absent – D. Late			352.22	−0.86 [-1.36, −0.38]	
R.A., D. Late – S. Late			0.75	−0.25 [−0.64, −0.02]	
Figure 5*B*					
L.A., D. Absent – D. Early			0.12	−0.08 [−0.31, 0.00]	
L.A., D. Early – S. Early			0.41	−0.19 [−0.54, −0.01]	
L.A., D. Absent – D. Late			0.06	−0.01 [−0.01, −0.01]	
L.A., D. Late – S. Late			0.14	−0.10 [−0.34, 0.00]	
R.A., D. Absent – D. Early			0.09	−0.07 [−0.26, 0.00]	
R.A., D. Early – S. Early			0.23	−0.13 [−0.45, −0.01]	
R.A., D. Absent – D. Late			0.05	0.00 [0.00, 0.00]	
R.A., D. Late – S. Late			0.18	−0.11 [−0.41, −0.01]	
Figure 5*C*					
L.A., D. Absent – D. Early			0.11	0.07 [0.00, 0.31]	
L.A., D. Early – S. Early			0.10	0.07 [0.00, 0.32]	
L.A., D. Absent – D. Late			62.01	0.71 [0.26, 1.18]	
L.A., D. Late – S. Late			0.12	0.08 [0.00, 0.31]	
R.A., D. Absent – D. Early			0.16	0.10 [0.01, 0.37]	
R.A., D. Early – S. Early			0.17	0.11 [0.01, 0.40]	
R.A., D. Absent – D. Late			2931.20	1.07 [0.55, 1.60]	
R.A., D. Late – S. Late			0.27	0.15 [0.01, 0.47]	
Figure 6*A*					
L.P., F. Absent – F. Early			0.13	−0.09 [−0.35, 0.00]	
L.P., F. Early – S. Early			0.51	−0.22 [−0.59, −0.02]	
L.P., F. Absent – F. Late			0.48	−0.21 [−0.56, −0.01]	
L.P., F. Late – S. Late			0.70	−0.26 [−0.64, −0.02]	
R.P., F. Absent – F. Early			0.25	−0.15 [−0.46, −0.01]	
R.P., F. Early – S. Early			0.22	−0.13 [−0.44, −0.01]	
R.P., F. Absent – F. Late			0.21	−0.13 [−0.44, −0.01]	
R.P., F. Late – S. Late			0.13	−0.09 [−0.35, 0.00]	
Figure 6*B*					
L.P., F. Absent – F. Early			2.85	−0.40 [−0.83, −0.06]	
L.P., F. Early – S. Early			0.19	−0.12 [−0.41, −0.01]	
L.P., F. Absent – F. Late			3.74	−0.44 [−0.86, −0.07]	
L.P., F. Late – S. Late			0.28	−0.16 [−0.49, −0.01]	
R.P., F. Absent – F. Early			0.10	−0.08 [−0.29, 0.00]	
R.P., F. Early – S. Early			0.20	−0.12 [−0.41, −0.01]	
R.P., F. Absent – F. Late			1.27	−0.33 [−0.74, −0.03]	
R.P., F. Late – S. Late			0.24	−0.14 [−0.47, −0.01]	

Figure 6*C*					
L.P., F. Absent – F. Early			1578.61	1.01 [0.50, 1.54]	
L.P., F. Early – S. Early			0.62	0.24 [0.02, 0.60]	
L.P., F. Absent – F. Late			8641.44	1.17 [0.62, 1.73]	
L.P., F. Late – S. Late			0.12	0.09 [0.00, 0.35]	
R.P., F. Absent – F. Early			15902.41	1.22 [0.67, 1.81]	
R.P., F. Early – S. Early			0.30	0.16 [0.01, 0.50]	
R.P., F. Absent – F. Late			4657.42	1.19 [0.63, 1.80]	
R.P., F. Late – S. Late			0.21	0.13 [0.01, 0.49]	
Figure 7*A*					
L.P., D. Absent – D. Early			0.37	−0.18 [−0.53, −0.01]	
L.P., D. Early – S. Early			0.39	−0.19 [−0.54, −0.01]	
L.P., D. Absent – D. Late			0.15	−0.10 [−0.38, 0.00]	
L.P., D. Late – S. Late			0.15	−0.10 [−0.38, 0.00]	
R.P., D. Absent – D. Early			0.12	−0.09 [−0.33, 0.00]	
R.P., D. Early – S. Early			0.13	−0.09 [−0.35, 0.00]	
R.P., D. Absent – D. Late			0.77	−0.26 [−0.64, −0.02]	
R.P., D. Late – S. Late			0.22	−0.13 [−0.44, −0.01]	
Figure 7*B*					
L.P., D. Absent – D. Early			0.36	−0.18 [−0.52, −0.01]	
L.P., D. Early – S. Early			0.17	−0.11 [−0.40, −0.01]	
L.P., D. Absent – D. Late			0.49	−0.21 [−0.58, −0.01]	
L.P., D. Late – S. Late			0.12	−0.08 [−0.33, 0.00]	
R.P., D. Absent – D. Early			0.29	−0.16 [−0.48, −0.01]	
R.P., D. Early – S. Early			0.20	−0.13 [−0.42, −0.01]	
R.P., D. Absent – D. Late			3.60	−0.43 [−0.85, −0.07]	
R.P., D. Late – S. Late			0.20	−0.12 [−0.42, −0.05]	
Figure 7*C*					
L.P., D. Absent – D. Early			5.19	0.47 [0.09, 0.90]	
L.P., D. Early – S. Early			0.18	0.12 [0.01, 0.41]	
L.P., D. Absent – D. Late			771.16	0.94 [0.45, 1.44]	
L.P., D. Late – S. Late			0.26	0.15 [0.01, 0.46]	
R.P., D. Absent – D. Early			110.27	0.76 [0.30, 1.24]	
R.P., D. Early – S. Early			0.24	0.14 [0.01, 0.46]	
R.P., D. Absent – D. Late			9,011,000.00	1.90 [1.18, 2.65]	
R.P., D. Late – S. Late			0.59	0.24 [0.02, 0.60]	

L.A., Left Anti; R.A., Right Anti; L.P., Left Pro; R.P., Right Pro; F., FEF cTBS, S., S1 cTBS, D., cTBS

## Results

### FEF versus control cTBS conditions: anti-saccades

#### Saccade amplitude

There was substantial evidence that FEF cTBS caused impairments in leftward anti-saccade amplitudes for conditions also involving PPC pulses, and for rightward anti-saccades for conditions involving the late PPC pulse (Table 3, Amplitude, BF_10_ > 3). There was not substantial evidence that the PPC pulse on its own produced an impairment, and there was also not substantial evidence (Fig. 4*A*, brackets, all BF_10_ ≤ 2.91) to indicate greater impairments from the double perturbation condition compared with either single perturbation condition.

**Table 3:** Bayes factors for the alternative (impairment) versus null (no impairment) hypothesis (BF_10_) for left and right anti-saccade trials relative to control cTBS

Left anti	cTBS site	PPC pulse	BF_10_	Right anti	cTBS site	PPC pulse	BF_10_
Amplitude	FEF	Absent	2.69		FEF	Absent	0.26
		Early	**4.09**			Early	1.31
		Late	**4.58**			Late	**7.60**
	S1	Early	0.35		S1	Early	0.52
		Late	0.39			Late	1.21
Percentage correct	FEF	Absent	0.15		FEF	Absent	0.16
		Early	0.06			Early	0.07
		Late	0.06			Late	0.06
	S1	Early	0.08		S1	Early	0.07
		Late	0.05			Late	0.06
SRT	FEF	Absent	0.10		FEF	Absent	0.13
		Early	0.27			Early	0.35
		Late	0.31			Late	1.39
	S1	Early	0.19		S1	Early	0.79
		Late	**2299.54**			Late	**2619.87**

Bold values: BF_10_ > 3. anti, Anti-saccade.

#### Percentage correct direction

There was not substantial evidence that anti-saccades were impaired by either form of TMS; in fact, strong evidence toward the null hypothesis was found for conditions with the PPC pulse (Table 3, Percentage correct, BF_10_ < 0.1). [Bayesian *t* tests performed in the opposite direction revealed substantial or greater evidence (BF_10_ > 3) for a performance benefit from the PPC pulses.] Similarly, there was strong evidence that there were not greater impairments from the double perturbation compared with either single perturbation (Fig. 4*B*).

#### Saccade reaction times

For SRTs, “decisive” (Wetzels et al., 2011) evidence for impairments were observed for conditions with the late PPC pulse alone, but not for those following FEF cTBS (Table 3, Saccade reaction time). Strong evidence was found that FEF cTBS plus a late PPC pulse resulted in greater impairments relative to FEF cTBS alone (and substantial evidence was found for a greater impairment for the early PPC pulse for leftward anti-saccades; Fig. 4*C*). However, strong evidence was found that impairments for leftward anti-saccades were not greater when the late PPC pulse followed FEF cTBS compared with when it was alone (Fig. 4*C*, BF = 0.08, italicized): when tested in the reverse direction, there was substantial evidence that the impairment after the late PPC pulse alone was greater than after FEF cTBS, BF_10_ = 4.12.

### DLPFC versus control cTBS conditions: anti-saccades

#### Saccade amplitude

**Figure 5.**
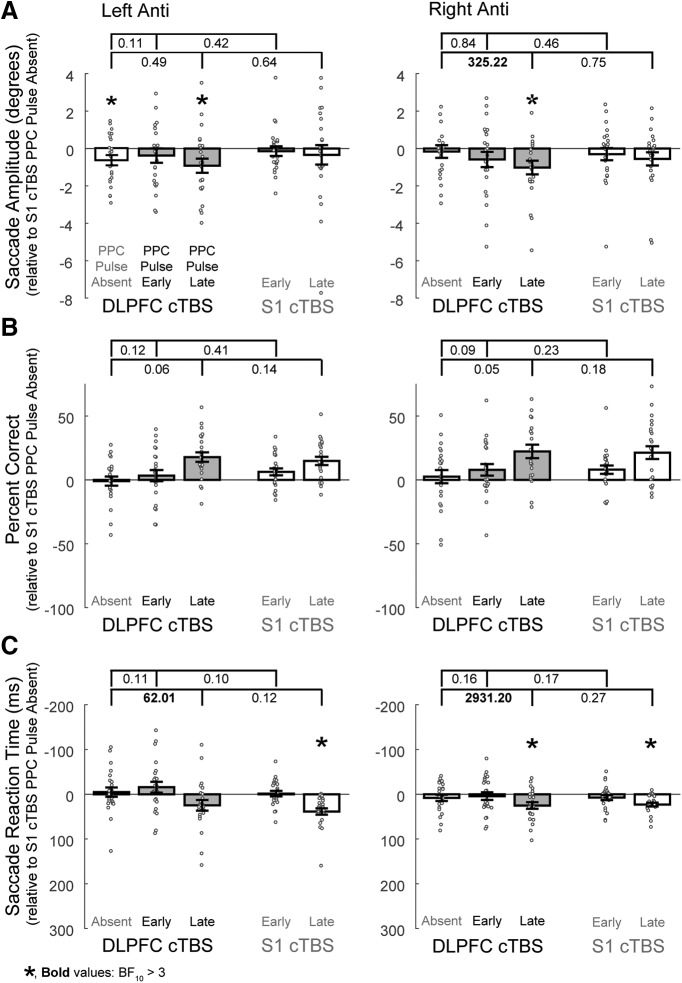
Effects on left and right anti-saccades when the double perturbation involving DLPFC cTBS and PPC TMS is compared with the single perturbation conditions for ***A***, Saccade amplitudes, ***B***, Percentage correct directions, and ***C***, Saccade reaction times. PPC pulse conditions relative to S1 cTBS are shown in duplicate as in Figure 4, and conventions are as in Figure 4.

There was substantial evidence for impairments to anti-saccades after DLPFC cTBS in conditions involving the late PPC pulse, and for DLPFC cTBS alone for leftward anti-saccades (Table 4, Amplitude). Strong evidence was found for a greater impairment from the combined perturbation effects for rightward anti-saccades after the late pulse relative to the DLPFC cTBS alone (BF_10_ = 325.22), but this was not found compared with the effects of the late PPC pulse alone (BF_10_ = 0.75; Fig. 5*A*).

**Table 4:** Bayes factors for the alternative (impairment) versus null (no impairment) hypothesis (BF_10_) for left and right anti-saccade trials relative to control cTBS (the effect of the PPC pulse relative to control cTBS is shown in duplication as in Table 3)

Left anti	cTBS site	PPC pulse	BF_10_	Right anti	cTBS site	PPC pulse	BF_10_
Amplitude	DLPFC	Absent	**4.21**		DLPFC	Absent	0.33
		Early	0.55			Early	0.98
		Late	**4.84**			Late	**8.84**
	S1	Early	0.35		S1	Early	0.52
		Late	0.39			Late	1.21
Percentage correct	DLPFC	Absent	0.27		DLPFC	Absent	0.16
		Early	0.14			Early	0.09
		Late	0.05			Late	0.05
	S1	Early	0.08		S1	Early	0.07
		Late	0.05			Late	0.06
SRT	DLPFC	Absent	0.16		DLPFC	Absent	0.68
		Early	0.11			Early	0.33
		Late	2.52			Late	**33.86**
	S1	Early	0.19		S1	Early	0.79
		Late	**2299.54**			Late	**2619.86**

Bold values: BF_10_ > 3. anti, Anti-saccade.

#### Percentage correct direction

There was no evidence that anti-saccades were impaired by DLPFC cTBS, with, or without, the PPC pulse (Table 4, Percentage correct). (Bayesian *t* tests revealed strong evidence for anti-saccade benefits to performance following DLPFC cTBS and late PPC pulses.) There was also no evidence for greater impairment from a double perturbation compared with single perturbation (Fig. 5*B*).

#### Saccade reaction times

There was strong evidence for impaired reaction times at the late pulse time following DLPFC cTBS for right anti-saccades (Table 4, Saccade reaction time), and there was strong evidence that the combined effects of DLPFC cTBS and a late PPC pulse resulted in greater impairments relative to DLPFC cTBS alone (Fig. 5*C*), but there was no evidence for greater impairment compared with the PPC pulse.

### FEF versus control cTBS conditions: pro-saccades

#### Saccade amplitude

**Figure 6.**
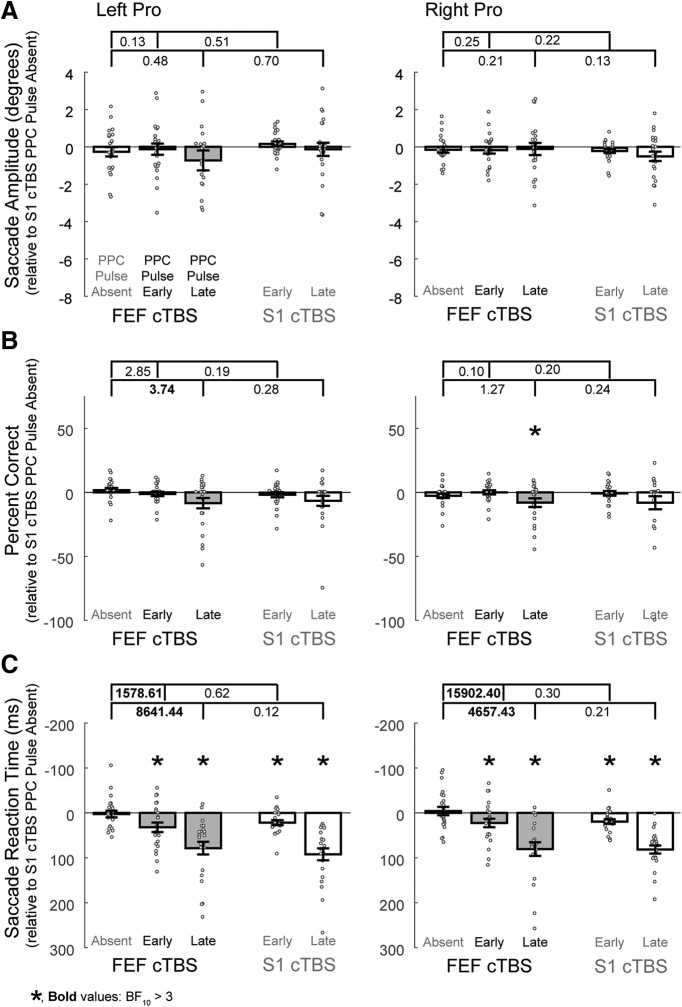
Effects on left and right pro-saccades when the double perturbation involving FEF cTBS and PPC TMS is compared with the single perturbation conditions for ***A***, Saccade amplitudes, ***B***, Percentage correct directions, and ***C***, Saccade reaction times. Conventions are as in Figure 4.

Table 5, Amplitude, and Figure 6*A* show that there was not substantial evidence for the effects of either TMS condition on pro-saccade amplitudes.

**Table 5:** Bayes factors for the alternative (impairment) versus **null (no impairment) hypothesis (BF_10_) for left and right pro-saccade trials relative to control cTBS**

Left pro	cTBS site	PPC pulse	BF_10_	Right pro	cTBS site	PPC pulse	BF_10_
Amplitude	FEF	Absent	0.61		FEF	Absent	0.54
		Early	0.31			Early	0.56
		Late	0.87			Late	0.30
	S1	Early	0.11		S1	Early	1.07
		Late	0.30			Late	2.32
Percentage correct	FEF	Absent	0.12		FEF	Absent	1.16
		Early	0.41			Early	0.22
		Late	2.63			Late	**4.53**
	S1	Early	0.48		S1	Early	0.24
		Late	1.44			Late	1.19
SRT	FEF	Absent	0.29		FEF	Absent	0.16
		Early	**14.88**			Early	**4.08**
		Late	**3314.92**			Late	**1461.64**
	S1	Early	**110.56**		S1	Early	**51.42**
		Late	**52,637.20**			Late	**2,165,000**

Bold values: BF_10_ > 3. pro, Pro-saccade.

#### Percentage correct direction

Substantial impairments were found for rightward pro-saccades following FEF cTBS during trials with the addition of a late PPC pulse (Table 5, Percentage correct, BF_10_ = 4.53). There was also substantial evidence that the impairments to leftward pro-saccades were greater following FEF cTBS when there was a late PPC pulse (Fig. 6*B*, BF_10_ = 3.74) compared with FEF cTBS alone. There was not substantial evidence for other impairments.

#### Saccade reaction times

Substantial or greater evidence for pro-saccade reaction time impairments was observed for all PPC pulse conditions (Table 5, Saccade reaction time). There was also strong evidence that the combined effects of FEF cTBS and PPC pulses resulted in greater impairments relative to FEF cTBS alone (Fig. 6*C*); however, there was no evidence for a greater impairment over the PPC pulse effects alone.

### DLPFC versus control cTBS conditions: pro-saccades

#### Saccade amplitude

**Figure 7.**
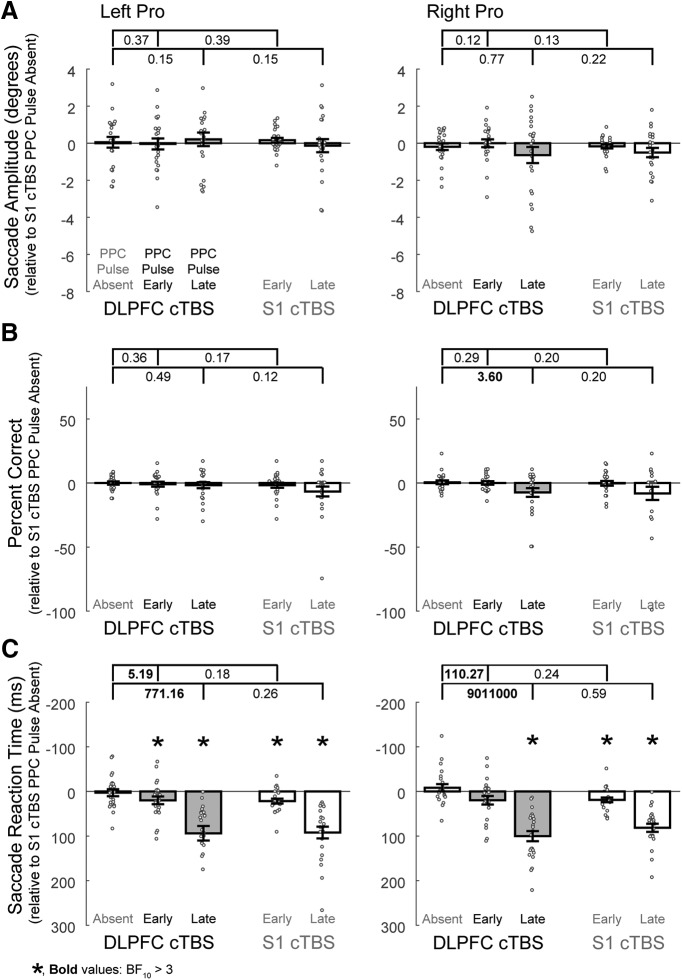
Effects on left and right pro-saccades when the double perturbation involving DLPFC cTBS and PPC TMS is compared with the single perturbation conditions for ***A***, Saccade amplitudes, ***B***, Percentage correct directions, and ***C***, Saccade reaction times. Conventions are as in Figure 4.

There was not substantial evidence for any effects to pro-saccade amplitudes (Table 6, Amplitude, Fig. 7*A*).

**Table 6:** Bayes factors for the alternative (impairment) versus **null (no impairment) hypothesis (BF_10_) for left and right pro-saccade trials relative to control cTBS (the effect of the PPC pulse relative to control cTBS is shown in duplication as in Table 5)**

Left pro	cTBS site	PPC pulse	BF_10_	Right pro	cTBS site	PPC pulse	BF_10_
Amplitude	DLPFC	Absent	0.19		DLPFC	Absent	0.62
		Early	0.24			Early	0.22
		Late	0.15			Late	1.03
	S1	Early	0.11		S1	Early	1.07
		Late	0.30			Late	2.32
Percentage correct	DLPFC	Absent	0.22		DLPFC	Absent	0.17
		Early	0.32			Early	0.21
		Late	0.42			Late	2.84
	S1	Early	0.48		S1	Early	0.24
		Late	1.44			Late	1.19
SRT	DLPFC	Absent	0.29		DLPFC	Absent	0.12
		Early	**3.65**			Early	2.49
		Late	**5089.32**			Late	**2,344,000**
	S1	Early	**110.56**		S1	Early	**51.42**
		Late	**52,637.20**			Late	**2165000**

Bold values: BF_10_ > 3. pro, Pro-saccade.

#### Percentage correct direction

There was substantial evidence that the impairments to rightward pro-saccades were greater following DLPFC cTBS when the late PPC pulse was present (Fig. 7*B*; BF_10_ = 3.60), but no other evidence for impairments was substantial (Table 6, Percentage correct).

#### Saccade reaction times

There was decisive evidence for reaction time impairments at the late PPC pulse time following DLPFC cTBS, and substantial evidence for impairments at the early PPC pulse time for leftward anti-saccades (Table 6, Saccade reaction time). Also, there was substantial evidence that the combined effects of DLPFC cTBS and PPC pulses resulted in greater impairments relative to DLPFC cTBS alone (Fig. 7*C*).

## Discussion

We found Bayesian evidence for impaired FEF and DLPFC anti-saccade amplitudes following a cTBS perturbation, and that compensation by PPC was possible after DLPFC cTBS perturbed ipsilateral anti-saccades. There was no evidence that cTBS impaired anti-saccade reaction times or correct directions, and we note that the impairments to anti-saccade amplitudes were not found in every condition following cTBS alone. Interestingly, however, we did not find any Bayesian evidence for an augmented effect, whereby the two TMS perturbations built on one another, suggesting instead the effects are generated at the network rather than nodal/regional level only.

Performance of pro-saccades and anti-saccades involves cortical and subcortical regions including FEF, PPC, DLPFC, supplementary eye fields, anterior cingulate cortex, visual cortex, basal ganglia, cerebellum, superior colliculus, and brainstem reticular formation (Moschovakis et al., 1996; Munoz and Everling, 2004; Munoz and Schall, 2004; Everling and DeSouza, 2005; Ford et al., 2005; Medendorp et al., 2006; Schall, 2009). A frontoparietal, precuneus, and parietal-medio-temporal network has also been identified as being involved in anti-saccade generation by independent company analysis-based fMRI, in addition to an eye-field network involved in both pro-saccades and anti-saccades (Domagalik et al., 2012). This highlights the wide-ranging involvement of several brain networks with the implication that one may not always observe deficits after a TMS perturbation or lesion, given the potential for redundancy or “degeneracy” (Price and Friston, 2002). Nevertheless, key neurophysiological processes related to voluntary saccade programming, reflexive saccade inhibition, and attentional reorienting processes point to important nodal roles for FEF, PPC, and DLPFC, explaining why deficits can result from single lesions or perturbations.

### The frontal eye fields

In FEF, “saccade” and “fixation” neurons could provide two substrates for saccade programming and saccade inhibition. First, some of the saccade neurons code for the motor goal of saccades, while others process visual and visuomotor information (Bruce and Goldberg, 1985; Schlag-Rey et al., 1992; Schall, 2002; Sato and Schall, 2003; Schall et al., 2011). Reversible FEF lesions by cooling probe in monkeys were shown to produce hypometria (Keating and Gooley, 1988; Peel et al., 2014), and patients with FEF lesions have shown reduced contralateral saccade amplitudes (Rivaud et al., 1994; Ploner et al., 1999), though not always (Terao et al., 2016). Second, FEF saccade neurons show decreased activity during the preparatory phase of anti-saccade compared with pro-saccade trials (Everling and Munoz, 2000). FEF fixation neurons, on the other hand, show increased activity during fixation (even in the absence of a stimulus; Hanes et al., 1998; Izawa et al., 2009), implying that they are substrates for stopping reflexive saccades (Munoz and Everling, 2004; Boucher et al., 2007; Schall and Godlove, 2012). Indeed, some patients with lesions encompassing FEF have shown difficulty in suppressing reflexive saccades (Guitton et al., 1985; Van der Stigchel et al., 2012; Terao et al., 2016), and increased voluntary saccade latencies (Terao et al., 2016). However, one patient with a highly circumscribed left FEF lesion showed no deficits in inhibiting reflexive saccades, but did have hypometria (Gaymard et al., 1999). Together, this shows that FEF is important to voluntary saccade programming, but task, or lesion, specifics may dictate whether its role is critical given the potential for the contributions from other network regions with neuronal populations that can carry similar information. Evidence shows, for instance, that deficits following an FEF lesion become more severe if the superior colliculus is also lesioned (Schiller et al., 1979; Keating and Gooley, 1988).

TMS perturbations to FEF have largely produced similar effects. Like lesions, TMS perturbations lack the specificity to affect saccade neurons uniquely from fixation neurons, meaning that caution should be taken in attempts to interpret the effects on particular neuronal populations. A single TMS pulse to FEF increased the latency for ipsilateral anti-saccade trials, but did not increase pro-saccade errors (Müri et al., 1991; Olk et al., 2006). However, in another study, a single TMS pulse to FEF at 100 ms post-stimulus onset, increased anti-saccade latency and increased the frequency of contralateral pro-saccade errors (Terao et al., 1998). (This distinction may be due to the fact that single pulses during anti-saccade generation would perturb an ongoing process whereby anti-saccade processes are in competition with more automatic pro-saccade signals, an effect that can explain our findings regarding pro-saccade and anti-saccade reaction times.) Another study showed increases latency in both pro-saccade as well as anti-saccade trials, particularly late during preparation (at 200 ms; Nagel et al., 2008). In a few studies, cTBS to FEF was shown to increase reaction times (Nyffeler et al., 2006a,b; Liu et al., 2011), but in other cases cTBS was reported to affect saccade amplitudes instead (Jaun-Frutiger et al., 2013; Cameron et al., 2015).

### The posterior parietal cortex

In monkeys, the generation of anti-saccades recruits lateral intraparietal area (LIP) neurons (the region of the primate PPC most associated with attention and eye movements; Gottlieb and Goldberg, 1999; Zhang and Barash, 2000; Bisley and Goldberg, 2010). LIP has been described as a “priority” map for attentional orienting, either overtly (a gaze change) or covertly (Bisley and Goldberg, 2010), integrating bottom-up visual information with top-down goal-directed information. Some LIP neurons signaling a visual stimulus then show activity during the motor component of vector inversion, which could be representing a remapped visual response (Zhang and Barash, 2000). In humans, PPC bilaterally (along with FEF) is shown to signal the vector inversion process (Medendorp et al., 2005; Moon et al., 2007; Collins et al., 2008). Patients with lesions to PPC have demonstrated saccade hypometria (Duhamel et al., 1992; Ptak and Müri, 2013), and those exhibiting neglect lesions often display erroneous saccades to ipsilesional “distractor” stimuli (Ptak and Müri, 2013) or deficits in remapping a saccade goal if the target changes position (Duhamel et al., 1992). Some patients display longer latencies on reaction times for reflexive, visually guided saccades (Pierrot-Deseilligny et al., 1991; Terao et al., 2016), fitting with evidence that the PPC may have a role in triggering express saccades (Hamm et al., 2010; Chen et al., 2013). Altogether, this highlights an important role of PPC in the visuo-motor aspects of saccade generation. Disruptive effects from TMS on these visuo-motor aspects is also consistent with these following observations: a TMS pulse to PPC shortly after stimulus onset (100 ms) produces hypometric anti-saccades to the ipsilateral (to TMS) direction, which then reverses to affect the motor vector in the opposite direction when applied later (>333 ms; Nyffeler et al., 2008b). Contralateral neglect is also reported from cTBS to right PPC (Nyffeler et al., 2008a).

### The dorsolateral prefrontal cortex

DLPFC is well known to be involved in cognitive control (Gazzaley and D’Esposito, 2007) and is therefore highly likely to be an important region in a network controlling voluntary saccades. Human and monkey studies have indeed found “preparatory” signals during pro-saccade or anti-saccade instruction periods in DLPFC (Everling and Munoz, 2000; Connolly et al., 2002; DeSouza et al., 2003; Everling and DeSouza, 2005; Ford et al., 2005; Brown et al., 2007), and SC neurons have been demonstrated to receive task-related signals from DLPFC (Johnston and Everling, 2006). There are also spatial signals in some DLPFC neurons, which is particularly important in visual working memory: DLPFC neurons were shown to have receptive/response fields with a contralateral bias (across the population) in working memory task delay periods (Funahashi et al., 1989; Ikkai and Curtis, 2011), which is not surprising if it shares information with FEF and PPC. Indeed, findings from human neuroimaging suggest that DLPFC is connected to FEF as well as to PPC functionally as well as anatomically (de Schotten et al., 2011; Vossel et al., 2014), and one physiologic study that recorded all three regions simultaneously in a sensorimotor decision task showed that sensory information “flows” from early visual regions to LIP, FEF, and DLPFC, and task-related signals flows from DLPFC and LIP to FEF (Siegel et al., 2015).

Patients with DLPFC lesions exhibit increased pro-saccade errors on anti-saccade trials (Guitton et al., 1985; Pierrot-Deseilligny et al., 1991; Ploner et al., 2005), suggesting it has a direct role in suppression. However, it has been difficult to dissociate a suppression role specifically of DLPFC from a role in task set establishment (Johnston and Everling, 2006; Johnston et al., 2009), as reflexive saccade errors following a DLPFC lesion could be explained by disruption to anti-saccade task-set signals to overcome the pro-saccade bias. A TMS pulse to DLPFC during the preparatory phase in an anti-saccade task did result in increased pro-saccade errors (Nyffeler et al., 2007), and “intermittent” TBS (thought to have excitatory effects; Huang et al., 2005) over DLPFC produced a reduction in pro-saccade errors (in patients with bipolar disorder; Beynel et al., 2014). In another study, however, a TMS pulse to DLPFC at the end of the preparatory period increased anti-saccade as well as pro-saccade latency, but not direction errors (Nagel et al., 2008).

TMS to DLPFC has also been shown to affect end point accuracy in memory saccades (Brandt et al., 1998), and DLPFC lesions resulted in higher variability in memory-guided saccade end points, with nonsignificant reductions in amplitudes (Pierrot‐Deseilligny et al., 2003), and a single-pulse TMS study did find that DLPFC pulses disrupted contralateral saccade amplitudes during the target memory component of a delayed saccade task (Müri et al., 1996). However, it has also been concluded in one lesion study that DLPFC was not necessary for performing the spatial calculations in a memory-guided saccade task (Mackey et al., 2016), and a study using cTBS to DLPFC did not find amplitude deficits to either ipsilateral or contralateral anti-saccades (Cameron et al., 2015).

### Implications from the double perturbation

As outlined above, individual lesion or TMS studies have indicated that FEF, PPC, and DLPFC are important to pro-saccade and anti-saccade tasks. However, there is a high level of variability across studies in the types of behavioral deficits one observes. This may be the result of relative unfocused effects of a TMS perturbation, or lesion, on the underlying populations, and/or network-level effects that extend beyond the role of an individual node. This implies that caution should be taken in assuming that any one TMS (or lesion) study can definitively define the role of an oculomotor region. In this study, we focus on the effects of a double perturbation compared with a single perturbation in a single paradigm and environment, acknowledging that the specifics of the paradigm may make direct comparisons with other studies difficult.

#### FEF versus control cTBS conditions: anti-saccades

We did not find evidence to suggest an augmented impairment effect (Hypothesis A) from the double perturbation across any of the saccade behaviors. Substantial evidence did suggest impairments to anti-saccade amplitude in FEF cTBS conditions when PPC pulses were present; however, because there was not substantial evidence that PPC pulses on their own caused impairments, nor were the effects greater following the double perturbation relative to following FEF cTBS alone, we conclude that cTBS to FEF on its own was consequential to anti-saccade amplitudes. We suggest that FEF cTBS had a “distributed” effect on processing in the network (Hypothesis B).

For saccade reaction times, we found evidence for greater impairments from the double perturbation compared with FEF cTBS on its own. The observation that a second perturbation produces a deficit that is not otherwise observed unless the first node is perturbed, is the argument to indicate compensation by that second node (Sack et al., 2005). We do not, however, believe our findings here indicate compensation by PPC (the second node), because the combined FEF cTBS plus PPC pulse conditions did not actually reveal substantial evidence for impairing behavior (Table 3, Percentage correct). In fact, the late PPC pulses on their own produced impairments that were greater than the double perturbation for contralateral anti-saccades (Hypothesis E). We conclude therefore that later PPC pulses were disruptive to the motor component of the anti-saccade. Following FEF cTBS, however, a compensatory mechanism might be revealed by other network structures that aid in anti-saccade generation. One possibility is that after FEF cTBS, there is compensation by DLPFC–colliculus projections to contralateral SC saccade neurons (Everling and Johnston, 2013), reducing the disruptive effect from a PPC pulse on the same network structures. This is sensible, considering the PPC pulses also produced substantial anti-saccade performance benefits in percentage correct directions, and human EEG evidence has shown that the posterior parietal/occipital cortex is involved in triggering express pro-saccades (Hamm et al., 2010), possibly by a cortical-SC mechanism (Watanabe et al., 2010; Chen et al., 2013). A PPC pulse could therefore disrupt the bias toward stimulus-driven saccades, thus indirectly facilitating anti-saccade performance.

Altered SC function could contribute to both the behavioral deficits, as well as to compensatory effects in either visuomotor or executive control for the following reasons: it receives widespread projections from the retina, subcortical, and cortical brain regions, including FEF, PPC, and DLPFC, and thus, its activity is influenced by the afferent signals it receives; it has a spatial map for programming a saccade to a particular spatial location; it has the internal architecture for directly translating visual information into the motor commands, which it also sends to the brainstem saccade generator circuits, and finally, it has fixation and saccade neurons, which could play a role similar to those described in FEF (Munoz and Everling, 2004; Munoz and Schall, 2004; Boucher et al., 2007; Watanabe and Munoz, 2011).

However, we acknowledge that these effects could be driven in part by the auditory/or somatosensory influence of the pulse (Duecker et al., 2013; Duecker and Sack, 2013), which could engage a startle-like reflex that inhibits ongoing motor commands by also acting on the SC or brainstem saccade generator circuits (Xu-Wilson et al., 2011; perhaps with less of a consequence in cases of compensation). As the goal of this study was to compare hypotheses regarding the double vs single perturbation situations, the important comparisons are those between the PPC pulses following control versus verum cTBS, which both have the same auditory/somatosensory influences of the PPC pulse.

#### DLPFC versus control cTBS conditions: anti-saccades

We found strong evidence for “compensation” by PPC (Hypothesis C) following DLPFC cTBS for ipsilateral (rightward) anti-saccade amplitudes, but not substantial evidence for an augmented effect (Hypothesis A). Importantly, there was not substantial evidence that the PPC pulses alone produced an impairment. This finding is consistent with a compensatory mechanism, in that the second perturbation impairs a node that has assumed a greater contribution (Sack et al., 2005; Hartwigsen et al., 2016). We note that these effects were lateralized, as compensation was seen only in this ipsilateral direction. cTBS to DLPFC alone produced impairments in the contralateral direction, suggesting that DLPFC perturbations were more consequential for contralateral anti-saccades. The finding on its own is interesting as it suggests that DLPFC may be part of the vector inversion process previously emphasized to involve FEF and PPC (Munoz and Everling, 2004; Medendorp et al., 2005; Moon et al., 2007). However, the mixed findings from previous TMS and lesion studies lend support to a hypothesis that the spatial calculations for anti-saccades are performed by a distributed process. We can only speculate that compensation occurs in some circumstances depending on the particular task demands, such as spatial working memory complexity.

As with FEF cTBS, there was no evidence that any of the conditions impaired the percentage correct direction, but there was evidence for greater SRT impairments from the combined double perturbation compared with DLPFC cTBS alone. As addressed, the late PPC pulse impaired SRT on its own, suggesting that the effects are more related to that of the PPC pulse.

#### FEF versus control cTBS conditions: pro-saccades

There was no evidence to suggest that TMS to FEF or PPC impaired pro-saccade amplitudes, suggesting that other regions in a wider network are sufficient for the spatial calculations for a pro-saccade (Munoz and Schall, 2004). There were findings to suggest that the late PPC pulses following FEF cTBS impaired pro-saccade correct directions and that PPC pulses substantially increased reaction times, suggesting a detrimental effect of the PPC pulse, possibly by impairing PPC–SC signals (as described previously). We acknowledge, however, that because we rejected trials when reaction time was less than the PPC pulse time, the outcome measures of the late PPC pulse are biased as coming from pro-saccade trials with a slower latency.

#### DLPFC versus control cTBS conditions: pro-saccades

As with FEF cTBS, DLPFC appears not to be critical to pro-saccade amplitudes. Interestingly, the late PPC pulse following DLPFC cTBS impaired rightward pro-saccade performance compared with DLPFC cTBS alone, but it is difficult to interpret this as compensatory as this condition did not actually produce substantial evidence for an impairment (BF_10_ < 3; Table 6).

### Conclusions

Our findings for a general lack of augmented effects from two TMS perturbations to critical nodes in anti-saccade programming suggest that these saccade behaviors are governed by distributed computations. Yet, if these regions are critical for behavior, how can we reconcile a lack of augmented effects from a double perturbation? Given evidence that anti-saccade vector inversion is developed simultaneously in FEF and PPC neuronal populations, our cTBS effects may be interpreted as being consequential for the communication of information between nodes (Sporns et al., 2007; Bullmore and Sporns, 2009) rather than for perturbing nodal computations only. FEF, DLPFC, and PPC are part of interconnected frontoparietal networks that are recruited when attentional control is needed (Dosenbach et al., 2008; de Schotten et al., 2011; Ptak, 2012; Vossel et al., 2014; Tschentscher et al., 2017). FEF and DLPFC may be critical nodes in terms of network-level processes, behaving as “connector hubs” for long-range information flow (Sporns et al., 2007; Bullmore and Sporns, 2009). A cTBS perturbation to FEF, or DLPFC, may therefore be consequential for the communication of information. Neuronal oscillations (not addressed in this study) nevertheless have been shown to be modulated in a cortical oculomotor network by TMS (Marshall et al., 2015) and could represent a “collective-order process” in network-level representations and interactions (Buzsáki, 2006, p 25). Together, this study illustrates how network interactions are important over summated contributions of individual nodes.

## Synthesis

Reviewing Editor: Bradley Postle, University of Wisconsin Decisions are customarily a result of the Reviewing Editor and the peer reviewers coming together and discussing their recommendations until a consensus is reached. When revisions are invited, a fact-based synthesis statement explaining their decision and outlining what is needed to prepare a revision will be listed below. The following reviewer(s) agreed to reveal their identity: René Müri.

Here is the full text of the two reviews:

R#1:

This study investigates the effects of single-pulse TMS over the PPC of humans performing a pro-/anti-saccade task following either continuous theta-burst stimulation (cTBS) over the FEF or the DLPFC. More specifically, the authors tested whether double stimulation protocols (cTBS + TMS pulse) have additive effects on anti-saccade performance: saccade amplitude and latency as well as percent trial correct. Neither single nor double stimulations were found to be significant, and the effects of double stimulations were generally indistinguishable from the effects of single stimulations alone. The authors conclude that anti-saccade production rests on neural processing distributed across the fronto-parietal cortical network.

In its current form, the manuscript is difficult to read, because of the long descriptions of the many experimental conditions. The schematics in Figure 1 somehow do not really help interpreting the results. It is also strange that the authors opted to test the additive-effect hypothesis without calculating predictions from the single stimulations. Using the Baysesian statistic to test significance from the null hypothesis is fine, but to to test the additive-effect hypothesis one must test whether the effects of double stimulations are significantly different from the sum of the effects of the single stimulations (and not from the effects of each single stimulation). Where the authors see evidence for some compensation by PPC following cTBS over DLPFC (see abstract and Fig 5A, right panel), I see rather that the impairment following the combined cTBS + TMS pulse approximates the sum of the impairments associated with the single stimulations. The effects of FEF cTBS + PPC TMS pulse also appear to approximate the sum of the impairments associated with the single stimulations (Fig 4A, both panels). Some modeling is necessary to adequately interpret these data.

On another note, it is unclear whether this study's findings generally replicate previous work, in which cTBS was applied to single node of the network (Nyffeler  et al. 2006; Liu et al. 2011; Jaun-Frutiger et al. 2013; Cameron et al. 2015; Gurel et al. 2018; see also Lowe et al. 2018 for review as well as work in nonhuman primates: Gerit et al. 2011; Balen et al. 2017). In addition, this study's findings must also be reconciled with findings of previous single-pulse TMS studies using the anti-saccade paradigm (Muri et al. 1991;Terao et al 1998; Nagel et al. 2006; Olk et al. 2006; Nyffeler et al., 2007; Juan et al. 2008; Nyffeler  et al., 2008; Akaishi et al. 2010; Kunita & Fujiwara 2013; see also work in nonhuman primates: Valero-Cabre et al. 2012). In other words, the manuscript fails to adequately review the relevant literature.

It is understood that there can be significant differences between TMS studies, simply because there is considerable variability in the responses of healthy individual's to cTBS (Huang et al. 2005; Jannati et al. 2017) and single TMS pulse (Maeda et al. 2000). The authors should considered reporting individual data, together with mean and variance, in their summary figures (4-7).

Considering that the production of an anti-saccade requires at least a few neural processes, and a cortical network that extends beyond the 3 nodes considered in this study (Ettinger et al. 2008; Domagalik et al. 2012; Talanow et Ettinger 2016), the manuscript must discuss the substrates of some of these processes. For instance, there is solid evidence from human and nonhuman primate studies that the PPC plays a role in visual vector inversion (Everling et al. 1997; Medendorp et al. 2005; Nyffeler et al. 2007; Zhang & Barash, 2000, 2003; Herweg et al. 2014; see also work in nonhuman primates: Sanders et al. 2010). Could the lengthening of anti-saccade latency (and the associated increase in percent correct) following PPC TMS pulses be interpreted as the result of a compromised vector inversion process? And would the observation that the FEF cTBS does not change saccade latency and accuracy argue against the suggestion that the FEF is also implicated in vector inversion (Jaun-Frutiger et al. 2013)? Phosphenes, which cab be evoked from PPC TMS (Fried et al 2011), could also induce interference. The manuscript's Discussion should integrate better the several findings of the study.

Lastly, contextual (sequential) effects on saccade latency are well known, such as the latency of an anti-saccade depends on the type of trial previously performed (pro- versus anti-) as well as whether it was correct or incorrect (Akaishi et al. 2010; DeSimone & Health 2014). This is an important variable to control, especially because it can impact subjects differently.

R#2:

This is an interesting and well-written manuscript dealing with the problems of TMS effects on brain function. The study addresses the question whether TMS may affect at network-level rather than at nodal-level processes. They examined the effects of a double stimulation using TBS and single pulse TMS in the fronto-parietal networks. I like this elegant study, however, I have several major concerns.

Major concerns:

1) A general major concern is the use of the word “lesion” in context of TMS application. This is misleading and wrong, especially for single pulse TMS application. There are no studies showing a structural lesion of the cortex after rTMS application. TMS protocols have either an inhibitory or excitatory effect on behavior or neurophysiology such as MEP, but these effects are transient and therefore time-limited. The authors should find another wording.

2) A second major concern is the problem of effect and duration of TBS on behavior. They used the classical 50Hz TBS protocol of Huang et al. (2005). We now since the paper of Goldsworthy et al. (2012) that there are considerable individual differences of the cTBS effect on MEP (even reversing the inhibitory effect into an excitatory effect). It would be good that the authors discuss this point. Furthermore, for the duration of the TMS effect the authors cite a reference (Wischnewski and Schutter, 2015), which gives a review of publications taking the MEP as outcome. They estimate that cTBS effect lasts for 50 minutes. However, they stimulate outside M1 but over the FEF. There is sparse literature about TBS effect over the FEF. Nyffeler et al. (2006) showed that cTBS stimulation of the FEF increases saccade latency for about 30 minutes. The authors should have a look at their data, and control whether there is a difference in latency between the first and the second part of the experiment. There is not enough evidence that TBS has a similar long effect if applied over the motor area or a non-motor area.

3) The Discussion is not really more than a repetition of the results. I would expect more a discussion in the context of the role of the network and hubs. Finally, it is a pity that mainly monkey studies are cited and the papers from the Pierrot-Deseilligny group and others who studied on the same theme in humans are missing.

## Author Response

The effects of a TMS double lesion to a cortical network

---------------------------------------------

Synthesis of Reviews:

Computational Neuroscience Model Code Accessibility Comments for Author (Required):

N/A

Significance Statement Comments for Author (Required):

Please consider reframing the significance statement wrt the use of the word “lesion,” as emphasized in one of the two reviews. Perhaps using “perturbation,” or even just “stimulation” (or “target of cTBS”?) would accomplish this?

Answer:

We have changed “lesion” to “perturbation” through the manuscript.

Comments on the Visual Abstract for Author (Required):

N/A

Synthesis Statement for Author (Required):

Here is the full text of the two reviews:

R#1:

This study investigates the effects of single-pulse TMS over the PPC of humans performing a pro-/anti-saccade task following either continuous theta-burst stimulation (cTBS) over the FEF or the DLPFC. More specifically, the authors tested whether double stimulation protocols (cTBS + TMS pulse) have additive effects on anti-saccade performance: saccade amplitude and latency as well as percent trial correct. Neither single nor double stimulations were found to be significant, and the effects of double stimulations were generally indistinguishable from the effects of single stimulations alone. The authors conclude that anti-saccade production rests on neural processing distributed across the fronto-parietal cortical network.

In its current form, the manuscript is difficult to read, because of the long descriptions of the many experimental conditions. The schematics in Figure 1 somehow do not really help interpreting the results. It is also strange that the authors opted to test the additive-effect hypothesis without calculating predictions from the single stimulations. Using the Baysesian statistic to test significance from the null hypothesis is fine, but to to test the additive-effect hypothesis one must test whether the effects of double stimulations are significantly different from the sum of the effects of the single stimulations (and not from the effects of each single stimulation). Where the authors see evidence for some compensation by PPC following cTBS over DLPFC (see abstract and Fig 5A, right panel), I see rather that the impairment following the combined cTBS + TMS pulse approximates the sum of the impairments associated with the single stimulations. The effects of FEF cTBS + PPC TMS pulse also appear to approximate the sum of the impairments associated with the single stimulations (Fig 4A, both panels). Some modeling is necessary to adequately interpret these data.

Answer:

We have simplified the descriptions in Figure 1 to better emphasize the critical points of the hypotheses.

We agree with the reviewer that the term “additive” does not appropriately label the point of our hypothesis. We do not wish to test for precisely summated effects of the single stimulations. We are interested in effects that could be enhanced by the double perturbation, but need not equate the calculated predicted sum of the individual perturbations. Therefore, we have replaced “additive” with “augmented”.

On another note, it is unclear whether this study's findings generally replicate previous work, in which cTBS was applied to single node of the network (Nyffeler et al. 2006; Liu et al. 2011; Jaun-Frutiger et al. 2013; Cameron et al. 2015; Gurel et al. 2018; see also Lowe et al. 2018 for review as well as work in nonhuman primates: Gerit et al. 2011; Balen et al. 2017). In addition, this study's findings must also be reconciled with findings of previous single-pulse TMS studies using the anti-saccade paradigm (Muri et al. 1991;Terao et al 1998; Nagel et al. 2006; Olk et al. 2006; Nyffeler et al., 2007; Juan et al. 2008; Nyffeler et al., 2008; Akaishi et al. 2010; Kunita & Fujiwara 2013; see also work in nonhuman primates: Valero-Cabre et al. 2012). In other words, the manuscript fails to adequately review the relevant literature.

Answer:

We agree with the reviewer for this point and have added extensively to the Discussion (paragraphs 2 to 7 of the Discussion, in red) where we address the findings from previous lesion studies, TMS studies, as well as describing previous neurophysiological evidence for the roles of these regions in pro- and anti-saccade behaviors.

It is understood that there can be significant differences between TMS studies, simply because there is considerable variability in the responses of healthy individual's to cTBS (Huang et al. 2005; Jannati et al. 2017) and single TMS pulse (Maeda et al. 2000). The authors should considered reporting individual data, together with mean and variance, in their summary figures (4-7).

Answer:

We agree with the reviewer and have added single subject data onto Figures 4-7.

Considering that the production of an anti-saccade requires at least a few neural processes, and a cortical network that extends beyond the 3 nodes considered in this study (Ettinger et al. 2008; Domagalik et al. 2012; Talanow et Ettinger 2016), the manuscript must discuss the substrates of some of these processes. For instance, there is solid evidence from human and nonhuman primate studies that the PPC plays a role in visual vector inversion (Everling et al. 1997; Medendorp et al. 2005; Nyffeler et al. 2007; Zhang & Barash, 2000, 2003; Herweg et al. 2014; see also work in nonhuman primates: Sanders et al. 2010). Could the lengthening of anti-saccade latency (and the associated increase in percent correct) following PPC TMS pulses be interpreted as the result of a compromised vector inversion process? And would the observation that the FEF cTBS does not change saccade latency and accuracy argue against the suggestion that the FEF is also implicated in vector inversion (Jaun-Frutiger et al. 2013)? Phosphenes, which can be evoked from PPC TMS (Fried et al 2011), could also induce interference. The manuscript's Discussion should integrate better the several findings of the study.

Answer:

We have added details regarding the physiological substrates and the roles of DLPFC, FEF and PPC in executive function, saccade programming, and the vector inversion process (see Discussion paragraphs 3 to 7 under the headings “The Frontal Eye Fields”; “The Posterior Parietal Cortex”, and “The Dorsolateral Prefrontal Cortex”.

However, we are not sure how a disrupted vector inversion would relate directly to increase anti-saccade latency and increased-accuracy. Studies typically treat these as separate behaviors, due to differences in the underlying neuronal mechanisms, as discussed e.g., FEF fixation neurons which might have a role in suppressing reflexive saccades, would not have a clear role in vector inversion. In previous studies, Jaun-Frutiger et al. 2013 did not report measures on reaction time, and did not find any significant effects from cTBS to FEF on error rates, despite reporting hypometric ipsilateral anti-saccades, suggesting impairments to the visual component of the vector inversion process. Similarly, Nyffeler et al. 2008 did not report that error increased, but did report reduced gain after PPC TMS which corresponded with impaired vector inversion. We put forth an explanation about how the PPC TMS pulse can exert its effects differently on anti- compared to pro-saccades, based on the fact that pro-saccades are more automatic, and may involve a parieto-occipital-collicular mechanism that would be perturbed by a PPC pulse, ultimately altering the normal bias towards pro- compared to anti-saccade behavior.

Finally, with regards to Fried et al. 2011, they found phosphene thresholds over PPC at 61% of Maximal Stimulator Output (MSO) that is considerably higher than our mean MEP values derived at 43% of MSO, which we now report in the manuscript (albeit Fried used a Magstim 70mm device). Deblieck at al. 2008 found phosphene thresholds (over occipital cortex) to be 17% higher than AMT, and only 21/27 subjects could reliably perceive phosphenes. None of our subjects reported phosphenes.

Lastly, contextual (sequential) effects on saccade latency are well known, such as the latency of an anti-saccade depends on the type of trial previously performed (pro- versus anti-) as well as whether it was correct or incorrect (Akaishi et al. 2010; DeSimone & Health 2014). This is an important variable to control, especially because it can impact subjects differently.

Answer:

We are aware of the influence of previous trial effects (see also Barton et al. 2006). However, our experimental design does not allow for a reliable estimation of those sequential effects, given the variability of when the TMS pulse occurred, for instance, on a previous trial in the early (visual) phase, in the late (more motor) phase, or not at all. Note also that Akaishi et al. 2010 did not find that a single TMS pulse to FEF (set consistently at 400 ms after stimulus onset) affected behavior directly.

R#2:

This is an interesting and well-written manuscript dealing with the problems of TMS effects on brain function. The study addresses the question whether TMS may affect at network-level rather than at nodal-level processes. They examined the effects of a double stimulation using TBS and single pulse TMS in the fronto-parietal networks. I like this elegant study, however, I have several major concerns.

Major concerns:

1) A general major concern is the use of the word “lesion” in context of TMS application. This is misleading and wrong, especially for single pulse TMS application. There are no studies showing a structural lesion of the cortex after rTMS application. TMS protocols have either an inhibitory or excitatory effect on behavior or neurophysiology such as MEP, but these effects are transient and therefore time-limited. The authors should find another wording.

Answer:

We agree with this point, and have changed the word to “perturbation”.

2) A second major concern is the problem of effect and duration of TBS on behavior. They used the classical 50Hz TBS protocol of Huang et al. (2005). We now since the paper of Goldsworthy et al. (2012) that there are considerable individual differences of the cTBS effect on MEP (even reversing the inhibitory effect into an excitatory effect). It would be good that the authors discuss this point. Furthermore, for the duration of the TMS effect the authors cite a reference (Wischnewski and Schutter, 2015), which gives a review of publications taking the MEP as outcome. They estimate that cTBS effect lasts for 50 minutes. However, they stimulate outside M1 but over the FEF. There is sparse literature about TBS effect over the FEF. Nyffeler et al. (2006) showed that cTBS stimulation of the FEF increases saccade latency for about 30 minutes. The authors should have a look at their data, and control whether there is a difference in latency between the first and the second part of the experiment. There is not enough evidence that TBS has a similar long effect if applied over the motor area or a non-motor area.

Answer:

We agree with this reviewer on the importance of assessing individual variability in the duration of cTBS effects on behavior when that intervention is applied to non-motor areas. As one way to address variability, we now show the individual subject data in each of the figures.

Also, to address whether there were potential wearing-off effects in analyzing data up to 50 minutes post cTBS, we performed a multivariate repeated measures ANOVA of PPC pulse absent trials split into the first and second half across the three parameters of interest (saccade amplitudes, correct directions and reaction times). We did not find significant interactions between cTBS Site and Stimulation epoch (first half, second half; P > 0.18). This analysis is included in the manuscript on page 11.

Finally, we list here several reasons to base our study on a 50 minute estimate of effects:

besides the Wischnewski and Schutter's meta-analysis, another meta-analysis examining the effects from MEP-based cTBS in studies on DLPFC (Lowe et al. 2018) found an overall effect of inhibition from cTBS (though time was not mentioned in all studies); second, we (and others) have based our cTBS parameters on the more monitored MEP threshold for safety concerns (i.e., 80% of AMT), which is favored by our ethics board as it follows a standard protocol; thirdly, Hubl (also a co-author with Nyffeler) reported BOLD fMRI effects after FEF cTBS that did last over 60 minutes in some nodes (albeit not in all nodes, and with 8 subjects and a slightly different cTBS rhythm of 30 Hz); finally, analyzing data for 50 minutes rather than 30 minutes provides more trials to derive our Bayesian estimates.

3) The Discussion is not really more than a repetition of the results. I would expect more a discussion in the context of the role of the network and hubs. Finally, it is a pity that mainly monkey studies are cited and the papers from the Pierrot-Deseilligny group and others who studied on the same theme in humans are missing.

Answer:

We have expanded the Discussion, elaborating on lesion work and previous TMS studies (see the comments to reviewer 1). As suggested here by reviewer 2, we have also offered more insights into the mechanisms at the network level throughout the Discussion (especially in the first 7 paragraphs) and in the Conclusion.
